# Reducing patient waiting times in operating theatres via Lean 4.0: a qualitative study

**DOI:** 10.1108/IJHCQA-01-2026-0005

**Published:** 2026-06-05

**Authors:** Azhagu Poornima Karunakaran, Jiju Antony, Olivia McDermott, Micheal Sony, Andrea Sutoova, Arshia Kaul, Ranjit Roy Ghatak, Sercan Demir, Dewan Islam, Cristina Ciliberto

**Affiliations:** Newcastle University Business School, Newcastle Upon Tyne, UK; Northumbria University, Newcastle, UK; College of Science and Engineering, University of Galway, Galway, Ireland; Oxford Brookes University, Oxford, UK; Department of Quality Management, VSB-Technical University of Ostrava, Ostrava, Czech Republic; Faculty of Business and Economics, University of Melbourne, Melbourne, Australia; IMI Bhubaneswar, Bhubaneswar, India; Department of Operations, Northumbria University Newcastle, Newcastle Upon Tyne, UK; University of Messina, Messina, Italy; Northumbria University, Newcastle Upon Tyne, UK

**Keywords:** Patient waiting time, Healthcare sector, Operational process, Process inefficiency, AI, Lean 4.0

## Abstract

**Purpose:**

The purpose of this study is to improve the operating theatre workflow in the healthcare sector by integrating Lean and Industry 4.0 AI-based approaches.

**Design/methodology/approach:**

A qualitative methodology was conducted, involving operational and management professionals selected through purposive sampling, based on predefined inclusion criteria. Thematic analysis was conducted to analyse the data, supported by evidence from a prior scoping review.

**Findings:**

The study identified key delays in operating theatres, including late patient arrivals, poor team coordination, incomplete pre-operative preparations and inadequate pre-surgery assessment, but AI tools can predict delays with substantial accuracy, enabling proactive scheduling adjustments. The integration of AI and Lean was found to streamline OT workflows, which reduces delays in patient waiting time in healthcare.

**Research limitations/implications:**

A limitation of this study is a lack of empirical application of Lean and AI, and a current integration gap requires stronger staff training and involvement. However, the study has implications for practice in its recommendations to implement AI and Lean to improve patient flow.

**Originality/value:**

While Lean 4.0 is gaining popularity in general healthcare management, there remains a paucity of empirical research specifically targeting the intersection of AI- and Lean principles in the operating theatre. This study seeks to bridge that gap by synthesizing expert insights with existing literature to provide a focused framework for implementation.

## 1. Introduction

Patient operational processes are cross-departmental systems that strongly affect care quality and healthcare resource utilisation (Rosa *et al.*, 2023). Waiting times vary depending on whether a hospital is private or public, with public waiting times being higher and potentially affecting patient safety, morbidity and hospital operational efficiency (McDermott *et al.*, 2022).

Lean methodologies eliminate waste, reduce bottlenecks and optimise workflows to minimise patient waiting time by 50% in operating theatres (Souza *et al.*, 2021). Many studies have demonstrated the application of Lean in Healthcare to reduce waiting times, improve theatre flow and eliminate non-value-added activities for patients (Rosa *et al.*, 2024; McDermott *et al.*, 2022).More recently, the advent of digitalisation, also known as Industry 4.0, has led to the adoption of tools such as AI-driven predictive analytics, which have optimised patient flow and theatre scheduling (Khare *et al.*, 2025).

Artificial Intelligence (AI) in emergency and operating theatre settings has shown strong potential in predicting waiting times and optimising processes (Li *et al.*, 2022). Similarly, the lean approach enables hospitals to focus on patient value while reducing operational delays (Hammoudeh *et al.*, 2021).

Existing studies have primarily explored general waiting-time reduction strategies; however, limited research has focused on integrating Lean and Artificial Intelligence solutions to enhance both patient satisfaction and operational efficiency in operating theatres. While Lean can reduce non-value-added waste, digitalisation tools, when deployed in a Lean environment, can further reduce or, in some cases, eliminate non-value-added waste (Powell, 2024). This study examines how integrating Lean and Industry 4.0 methods or Lean 4.0, including Artificial Intelligence, can reduce patient waiting times, particularly in operating theatres (OTs). It aims to understand how this combination can improve efficiency, reduce patient waiting times in OT workflow and support better decision-making in surgical care operations and thus improve patient waiting times.

## 2. Literature review

**Figure 1:**
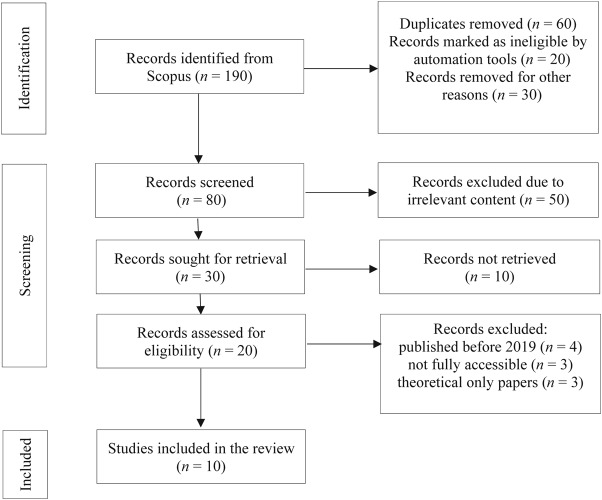
Prisma flow diagram

A scoping review was conducted to map the existing literature on the use of Lean Management and Artificial Intelligence (AI) in healthcare. To ensure relevance and high quality, articles published between 2019 and 2025 and indexed in the Scopus database were included. Studies were excluded if they were unrelated to Healthcare, published in languages other than English, or limited to purely theoretical papers without practical application. Earlier studies primarily examine Lean as a standalone approach and therefore fall outside the scope of this research. Following the defined criteria, the selection process was summarised using a PRISMA flow diagram (Figure 1).

Table 1 presents the data extraction and key findings.

**Table 1:** Literature related to research themes

Author	Study focus	Lean/AI/I4.0 element	Key contribution to waiting time reduction	Key limitations	Relevance to this study
Bachelet *et al.* (2019)	Interventions to reduce elective surgery waiting times	Lean pathways, scheduling optimisation	Demonstrates need for pathway redesign, prioritisation and OR planning	Variability across settings	Provides baseline Lean pathway optimisation logic
Mahmoud *et al.* (2021)	Impact of Lean management on staff and performance	Lean management	Shows reduction in waiting times and improved teamwork and learning	Risk of work intensification	Confirms Lean effectiveness in reducing delays
Botha *et al.* (2024)	AI risks in healthcare delivery	AI governance and safety	Highlights implementation risks affecting adoption of AI-driven scheduling	Conceptual and policy-oriented	Identifies barriers to AI integration in Lean systems
Stafinski *et al.* (2022)	Policy and operational approaches to surgical wait times	Lean/Six Sigma + system redesign	Shows effectiveness of standardised pathways, funding models and process redesign	Context-specific policy variation	Supports system-level Lean interventions
Dawson *et al.* (2022)	Clinical outcomes of prolonged waiting time	Outcome-based healthcare analysis	Demonstrates negative impact of long waiting times on patient outcomes	Measurement inconsistency	Strengthens urgency of reducing OT waiting time
Ebrahim *et al.* (2025)	Rural surgical access delays	Workforce and system coordination	Shows structural causes of delays and need for integrated planning	Rural context limitations	Highlights importance of resource coordination
O'Rielly *et al.* (2021), Strömblad *et al.* (2021)	Predictive models for surgical duration	AI predictive analytics	Improves surgical scheduling accuracy and OT utilisation	Limited real-time integration	Demonstrates AI role in OT scheduling optimisation
Abdallah and Alkhaldi (2019)	Lean with JIT and TQM in healthcare	Lean + JIT + TQM	Improves equipment efficiency and reduces operational delays	Limited digital integration	Provides Lean operational efficiency base

### 2.1 Summarising and reporting

The inefficiency of processes in the healthcare sector, associated with waiting times in the context of the operating theatre workflow, negatively affects patient satisfaction (Bachelet *et al.*, 2019). Prolonged waiting times can be associated with reduced patients' functional ability and quality of life (Dawson *et al.*, 2022). Among the factors contributing to wait times are insufficient hospital capacity and a lack of beds, financial constraints, variability in processes and lack of standardisation, insufficient operating theatre scheduling, ineffective resource utilisation, quality and data access, etc. (Stafinski *et al.*, 2022). Moreover, in rural areas, structural factors such as staff shortages, geographical distance and infrastructural limitations contribute to the issue, as well as others, including deviations from guideline-concordant care (Ebrahim *et al.*, 2025). According to Stafinski *et al.* (2022), the combination of different approaches is required, such as targeted funding, process improvement by Lean/Six Sigma implementation, standardised clinical pathways, patient prioritisation system, etc. The studies highlight the factors contributing to prolonged waiting times that are system-level, structural and organisational.

Lean approaches and AI can specifically target organisational factors by improving workflow efficiency, reducing process variability and enhancing resource allocation, while systemic barriers require broader policy-level interventions. According to Stafinski *et al.* (2022), the implementation of lean approaches can minimise patient waiting times by recognising customer value and reducing waste. Lean practices ensure resource efficiency by optimising staff, protective equipment and bed use to meet the requirements of urgent cases (Mahmoud *et al.*, 2021). In this regard, LM in Healthcare enhances the capacity to rapidly redesign and reallocate resources, thereby better aligning patient demand with system capabilities. Lean approaches, such as Just-in-Time (JIT) and Total Productive Maintenance (TPM), are effective in managing resources and reducing patient wait times (Abdallah and Alkhaldi, 2019).

AI supports centralised scheduling, effective operating theatre utilisation, and patient flow optimisation (Bachelet *et al.*, 2019). The study conducted by Strömblad *et al.* (2021) highlighted the promising potential of machine learning applications in predicting surgical durations and resource utilisation based on various types of historical data captured. The study by Hamza *et al.* (2025) focused on the effectiveness of utilising AI for automated skill assessment during surgeries, concluding that it can differentiate between various levels of surgical expertise by analysing operative video recordings using computer vision and deep learning. It means that AI helps to optimise the allocation of resources and predict the demands of the healthcare workflow based on this data. Despite the presented benefits of the AI applications. While Lean approaches and AI tools each contribute to reducing waiting times and optimal resource utilisation, their successful impact ultimately depends on addressing implementation challenges. Most applications have been fragmented and limited in scope. Issues of governance, workforce readiness and infrastructure are challenges that require attention. Based on the review, there is a limited understanding of the strategies applied by healthcare providers to efficiently implement AI-driven tools and lean approaches in reducing patients' waiting time in real-world, resource-constrained healthcare environments. These knowledge gaps will be addressed in this study by focusing on assessing real-time insights from health professionals regarding the usage, challenges and combined implementation of AI-driven tools and Lean approaches to enhance patient flow optimisation and reduce waiting times. Studies were assessed based on methodological rigour, strength of evidence, relevance to Lean 4.0, applicability to operating theatre waiting time reduction and technological maturity. Based on these criteria, studies were categorised as high, moderate or exploratory quality. Lean-focused studies generally demonstrated stronger empirical rigour, whereas AI-related studies were more exploratory and emerging. This appraisal enabled a more balanced and critical synthesis of the evidence base.

## 3. Research methodology

The study used inductive research approach, a key advantage of the inductive approach is its inherent flexibility, which facilitates the investigation of complex issues without being constrained by rigid theoretical assumptions, thereby allowing the research to adapt to the findings as they emerge (Azungah, 2018). This characteristic is crucial for exploring the multifaceted nature of operating theatre workflow disruptions. The inductive approach facilitates a deeper engagement with empirical evidence, ensuring that conclusions are grounded in authentic, context-specific data (Behal, 2023). To mitigate the potential limitation of reduced replicability, this study implemented rigorous and transparent documentation of all procedural steps. This research adopts a mono-method qualitative research choice, utilising qualitative data exclusively. This approach involves leveraging a single type of data to address the research questions (Bryman, 2010). The inherent limitation of the mono-method regarding triangulation (Bryman, 2010) is mitigated through the application of rigorous thematic analysis and a transparent coding process, which enhances the trustworthiness of the findings.

### 3.1 Research strategy

A dual-strategy approach was adopted, incorporating primary qualitative data collection alongside a scoping review of the extant literature. The primary strategy involved conducting semi-structured interviews. Primary qualitative strategies are recognised as effective for exploring complex experiences, perceptions and contextual factors that are not readily quantifiable (Bazen *et al.*, 2021). Semi-structured interviews were selected specifically for their capacity to allow participants to articulate their perspectives in depth while affording the researcher the flexibility to probe emergent themes, thereby ensuring data richness and complexity (Rowley, 2012). The interview guide was designed based on a literature review. The interview protocol was reviewed by all authors to ensure clarity, relevance and alignment with the research objectives. It was subsequently piloted with a small group of academic participants known to the research team, who possessed familiarity with the subject area. The scoping review was also conducted to complement and contextualise the primary data. This strategy provides a systematic overview of existing literature, aiding in the mapping of current knowledge, identifying conceptual gaps and strengthening the interpretive framework for the primary findings (Khalil *et al.*, 2025).

### 3.2 Sampling method

A purposive sampling technique was employed to recruit participants. This non-probability technique enables the deliberate selection of individuals most relevant to the research question (Campbell *et al.*, 2020), which was crucial for recruiting informants with direct experience in hospital operations and management. Participants were identified and recruited through targeted outreach on professional social media platforms, primarily LinkedIn. Study invitations were posted in relevant professional groups, and potential participants were also contacted directly if their profiles indicated experience in hospital operations. Interested individuals were provided with detailed information about the study, including objectives, inclusion criteria and ethical considerations, before confirming participation. Nine professionals were recruited based on predetermined inclusion and exclusion criteria (see Table 2). Data saturation (Francis *et al.*, 2010) was noted after 8, and hence it is concluded that the sample size is adequate for analysis. Data saturation has been noted in previous studies for a sample size of 8 or less, for example Morgan (2002) conducted a methodological study using qualitative data collected on environmental risks. They found that the first five to six interviews produced most themes. In a study the researchers interviewed 6 palliative health care professionals to study the perception of professionals in AI in palliative care (Ahmad *et al.*, 2025). In another study to understand midwives' perspectives regarding the effect of a programme of activities aimed at reducing alcohol exposed pregnancies six interviews were conducted with midwives (Morrello *et al*., 2022). These few studies are cited in order to stress the facts that data saturation is a subjective concept and this can be achieved with few sample size, and what matters is that sample size must be sufficient for key perceptions on a topic to be expressed (Malterud *et al*., 2016; Mason, 2010).The sample, though relatively homogeneous, consisted of information-rich participants with direct hospital operations experience.

**Table 2:** Participant inclusion and exclusion criteria

Criteria	Inclusion criteria	Exclusion criteria
Age	Aged 25–55 years	Individuals below 25 years or above 55 years
Industry relevance	Individuals from the healthcare industry with Healthcare operational exposure	Individuals from unrelated industries without Healthcare operational exposure
Experience	Minimum 2 years of professional experience in hospital operations	Less than 2 years of professional experience in hospital operations
Job profile	Hospital operational and management professionals	Non-hospital staff and clinical interns

The recruitment process involved disseminating online advertisements on various social media platforms used by professionals, such as popular sites like LinkedIn (Power, 2015). Institutional ethics committee approval was obtained prior to data collection, ensuring that all research procedures adhered to ethical guidelines and participants provided informed consent.

### 3.3 Data analysis

The collected qualitative data were analysed using a thematic analysis framework, as operationalised by Braun and Clarke (2006). This method is adept at identifying, analysing and reporting patterns (themes) within qualitative data, facilitating the generation of findings grounded in the empirical material. This systematic process was applied to both the interview transcripts and the literature from the scoping review to ensure a robust, integrated analysis.

## 4. Results

Following Braun and Clarke (2006) six-phase thematic analysis, we progressed from familiarisation to initial coding, theme construction, review, definition and integrative synthesis. In line with Braun and Clarke (2006), we began by repeatedly reading the interview transcripts to gain a holistic understanding of operating theatre processes and their pain points. Iterative immersion sensitised the analysis to recurrent operational frictions human-resource shortages, technological barriers and cultural resistance to change as well as to the roles that Lean 4.0 can play in improving patient flow, resource allocation and adjacent coordination tasks.

We collected data through semi-structured interviews that elicited detailed, context-rich accounts of the focal phenomenon. Interview transcripts were subjected to a systematic, multi-cycle coding procedure in which salient phrases, concepts and patterned actions were identified and assigned first-order codes. In the final stage, we abstracted overarching themes that captured the dominant, cross-cutting patterns in the dataset and articulated their explanatory links to the research questions. This thematic structure synthesises the core insights from participants' narratives and provides a coherent basis for interpretation. Table 3 presents the resultant themes.

**Table 3:** Resultant themes

Generated themes
*Theme 1:* Technological barriers, shortage of in-house expertise and process inefficiencies are causing delays in operational theatre service
*Theme 2:* Leveraging AI through machine learning, real-time analytics and predictive analytics to improve OT efficiency, optimise theatre utilisation to obtain proactive decision-making and minimise downtime
*Theme 3:* Integration of Lean tools such as Kanban, 5 S, Value stream mapping and Kaizen, along with AI-driven predictive analytics and real-time resource allocation to streamline OT workflows, optimise schedules and manage inventory to reduce delays
*Theme 4:* Lean and AI can be integrated to improve scheduling and monitoring through optimising shifts, anticipating cancellations and enabling data-driven decision-making by providing training to the staff

We conducted the theme review and interpretation in line with Braun and Clarke (2006) six-phase framework, aligning the emergent thematic structure with the study's objectives on reducing waiting times through Lean 4.0.

### 4.1 Theme 1: technological barriers, shortage of in-house expertise and process inefficiencies are causing delays in operational theatre service

Interview evidence indicates that multiple peri-operative inefficiencies compound to extend patient waiting times in operating theatres. Concurrent staffing shortfalls exacerbate these bottlenecks by limiting capacity to recover from disruptions, reallocate tasks or run parallel preparations. Together, these factors create cascading delays across the surgical pathway, increasing turnaround times and depressing theatre utilisation.

… Causes of delays in operating theatres are resource-related – particularly a shortage of human resources and suboptimal utilisation of the resources that are available. -P2

Participant P2 locates the proximate causes of delay in both capacity shortfalls and inefficient deployment of existing resources. Staff absences and late arrivals constrain the ability to execute scheduled lists, creating knock-on bottlenecks in pre-operative assessment and preparation. In turn, diminished slack reduces the system's capacity to absorb disruptions, precipitating schedule slippage and extended turnaround times.

Many delays can be traced back to fragmented systems and poor coordination between departments.-P2

Beyond coordination failures, organisational and cultural resistance also impede the adoption of Lean 4.0 practices. Because AI tools reconfigure work routines and decision rights, they catalyse cultural shifts that staff may resist, especially where psychological safety and participatory change processes are weak (Murire, 2024). Realising the complementary value of Lean 4.0 therefore requires visible executive sponsorship, disciplined change management and targeted capability building (e.g. training in standard work, data-literate problem solving and cross-functional huddles). Taken together, the evidence suggests that Lean 4.0 integration relies on strategic clarity, sustained leadership commitment and a shared operational vision that aligns the interests of clinical, managerial and informatics stakeholders.

Participants consistently highlighted resource and workflow inefficiencies as major contributors to OT delays. While most agreed that human-resource shortages and fragmented processes slow operations, some noted that even well-staffed theatres experience bottlenecks due to poor coordination or resistance to standardised practices. These contradictions suggest that delays are not solely a capacity issue but are shaped by organizational culture, interdepartmental communication and leadership engagement. Hospitals with structured protocols and supportive management perceived fewer disruptions, indicating that context moderates the impact of structural inefficiencies.

While participants correctly identified human-resource shortages and interdepartmental fragmentation as the primary causes of delay, these empirical findings align with the Lean theory of “Muda” (Waste), specifically the wastes of “Waiting” and “Motion.” We thus, infer that Lean 4.0 must therefore prioritize social-technical coordination over purely technical scheduling fixes.

### 4.2 Theme 2: leveraging AI through machine learning, real-time analytics and predictive analytics to improve OT efficiency to minimise downtime

Interview evidence portrays AI as a catalytic capability for compressing avoidable delays in operating theatres. Predictive analytics applied to case-duration estimation, overrun risk and likelihood of cancellation enables proactive list reshaping, earlier escalation and dynamic resource reallocation, thereby minimising idle time and improving theatre utilisation. As one participant noted:

I worked on predicted surgical delays with 85% accuracy, allowing proactive rescheduling to minimise patient wait times.*-P2*

The 85% predictive accuracy reported by P2 provides empirical evidence for what (Strömblad *et al*., 2021) theoretically propose as “predictive scheduling”. We differentiate our finding by noting that while the literature focuses on the mathematical duration of cases, our participants emphasize the behavioural value of “proactive rescheduling” to maintain psychological safety among staff. This implies that AI's primary contribution in this context is not just data precision, but “Decision Support”, which enables Lean “standard work” to remain flexible in high-variability environments like the OT.

In the aggregated narratives, AI tools function as decision support systems that surface emerging risks early enough for managers and clinicians to act, amplifying the effects of Lean disciplines such as standard work, visual controls, and daily huddles (Spence *et al.*, 2023). Together, these capabilities shorten turnaround intervals, decrease variability in start times and contribute to a sustained reduction in patient waiting times.

Artificial intelligence can help you to identify the root cause of problems. Artificial intelligence has been used to generate ideas concerning how to solve the situation.*-P3*

Participant P3 underscored AI's diagnostic and generative roles in addressing operating-theatre (OT) delays. In our data, AI tools particularly predictive analytics interrogate historical and real-time signals to surface recurrent delay patterns and propose targeted remedial actions. Predictive scheduling models estimate procedure durations based on patient characteristics, prior case histories and surgeon-level performance, thereby improving slot allocation and reducing downstream overruns. Complementing this, real-time analytics track theatre status (e.g. equipment readiness, instrument availability, staffing) and trigger early warnings for emerging bottlenecks. The resulting coordination gains help maintain patient flow across the surgical pathway, compress turnaround times and enhance overall operational efficiency.

AI was widely viewed as a catalyst for proactive decision-making, predictive scheduling and real-time monitoring. However, participants differed in their confidence in AI adoption: while some saw predictive analytics as transformative, others expressed concerns about data quality, integration challenges and staff readiness. These tensions reflect the interplay between technological potential and organizational readiness, emphasizing that AI benefits are contingent on workflow alignment, data completeness and technical capability.

### 4.3 Theme 3: integration of lean tools such as value stream mapping, 5S, Kanban and Kaizen with AI-driven predictive analytics to streamline OT workflows

Participant accounts referenced an implementation at the Mayo Clinic that combined Lean techniques specifically value stream mapping with AI-driven predictive scheduling to improve OT flow. As one informant reported:

They implemented value stream mapping to streamline OT workflows and used predictive analytics to optimise surgery schedules, reducing wait times by approximately 25%.*-P5*

This case study reported by P5 serves as a concrete example of Lean 4.0 in practice. There is a clear distinction here: the literature (Abdallah and Alkhaldi, 2019) primarily discusses Lean tools like Kanban and JIT as manual, physical systems. In contrast, our empirical findings suggest an evolution into “Digital Kanban”, where AI-driven resource allocation provides the real-time visibility that traditional Lean lacks. We infer that the “25% reduction in wait times” mentioned by P5 is a direct result of this technological-theoretical synergy. We treat this as an exemplar of complementary mechanisms: value stream mapping exposes non-value-adding steps and handoff frictions. At the same time, predictive scheduling refines case sequencing and duration estimates considering demand variability and resource constraints. According to P5's account, the coupled intervention yielded a substantial reduction in waiting times, consistent with the theorised synergy wherein Lean stabilises processes and AI enhances anticipatory decision-making.

… We use 5S so all instruments are kept in fixed labelled places*-P9*

Interviewees described systematic deployment of Lean instruments most prominently 5S and value stream mapping (VSM) to stabilise OT processes. Standardising locations and labelling for instruments (5S) reduced search time, shortened room-turnover activities and smoothed case preparation. At the same time, VSM exposed non-value-adding steps and handoff frictions that could be eliminated or redesigned. Consistent with prior evidence, participants linked these practices to fewer delays and more reliable, patient-centred flow.

Crucially, participants emphasised that AI capabilities amplify these Lean effects. AI-driven demand prediction informed Kanban/JIT parameters and staffing rosters. Real-time resource-allocation algorithms reconciled dynamic constraints across rooms, teams and equipment, and predictive models flagged impending shortages or bottlenecks early enough to trigger pre-emptive actions. Lean instruments (5S, VSM, Kaizen, Kanban) were reported to reduce delays and standardize processes, especially when coupled with AI predictive analytics. Contradictions emerged where Lean tools were implemented but staff adherence varied, or AI predictions were available but not acted upon due to hierarchical or cultural barriers. This highlights that the effectiveness of Lean 4.0 integration depends not only on the tools themselves but also on organizational practices, employee engagement and leadership support.

### 4.4 Theme 4: lean and AI improve scheduling and monitoring through optimising shifts, anticipating cancellations and enabling data-driven decision-making by providing training

Interviewees converged on a pragmatic sequencing for Lean 4.0 integration: begin with a tightly scoped pilot, demonstrate visible gains and then scale. As one respondent advised:

My advice is to start small. Choose one speciality and apply Lean basics first. Show results, so staff see the benefit.*-P8*

Recommendations prioritised low-risk pilots (e.g. clinic or OT scheduling) supported by targeted staff training, participatory planning and disciplined data hygiene. Early wins build confidence, help overcome cultural and organisational resistance and create momentum for staged adoption of Lean 4.0 capabilities.

The interviews underscored a set of recurrent impediments to reducing waiting times. Process inefficiencies – such as late arrivals, incomplete preoperative preparations, clinician unavailability and constrained staffing create cascading delays across the surgical pathway. Systemic factors compound these frictions: fragmented information flows, weak interdepartmental coordination during changeovers and the suboptimal utilisation of available resources. Cultural and organisational resistance further constrain adoption, particularly where AI requires shifts in routines, decision rights and accountability structures. Technological hurdles including incomplete datasets, data quality defects and limited in-house analytics capabilities also impede integration. Across accounts, respondents identified three key enabling conditions for successful Lean 4.0 deployment: a clear strategy and scope, visible leadership sponsorship and sustained capability building (including training in standard work, data-literate problem-solving and cross-functional huddles).

Participants consistently characterised AI as a catalytic layer that augments Lean disciplines. Predictive analytics help forecast cancellations and overruns, enabling proactive list resequencing and dynamic resource reallocation that reduce idle time and compress delays. Interviewees described models that estimate procedure durations using patient characteristics, surgeon performance histories and prior case data, improving slot allocation and on time starts. Real-time analytics monitor theatre status equipment readiness, instrument availability and staffing levels and alert teams to emerging bottlenecks for timely escalation. In tandem, AI capabilities assist with root-cause identification and option generation, supporting managers and clinicians in orchestrating smoother patient flow and more reliable throughput.

Respondents reported the routine use of foundational Lean tools. Value-stream mapping (VSM) illuminated non-value-adding steps and handoff frictions, while 5S standardised, labelled storage and layout reduced search time and accelerated preparations. Continuous-improvement routines (Kaizen) translated “small ideas” into measurable time savings (e.g. relocating trolleys to points of use, pre-staging IV lines). Interviewees also noted that AI amplifies Lean gains: demand forecasting informs Kanban/JIT parameters and staffing rosters; real-time optimisation engines reconcile room, team and equipment constraints and predictive alerts pre-empt shortages or schedule slippage. One illustrative case referenced by participants described a leading academic medical centre combining VSM with AI-driven predictive scheduling and achieving a substantial reduction in waiting times.

Across the dataset, the combined application of AI and Lean supports better shift management, equipment utilisation and scheduling accuracy thereby reducing downtime and sustained waiting times. Lean provides standardisation and waste reduction; AI enhances anticipation, visibility and rapid replanning. Participants emphasized phased implementation and training to optimise scheduling, anticipate cancellations, and enable data-driven decisions. While the value of Lean 4.0 was clear, some expressed concern that rapid implementation could overwhelm staff or generate resistance, particularly in hospitals with entrenched routines. These insights reveal that change management, staff involvement, and staged adoption are crucial for translating theoretical efficiency gains into operational reality.

The findings also highlight that Lean stabilizes processes while AI enables predictive scheduling and real-time resource allocation, reducing delays and optimizing theatre utilization. Context-specific contradictions emphasize that organizational alignment and leadership support are critical for successful Lean 4.0 implementation.

## 5. Discussion

The scoping review and findings underscore that significant process inefficiencies in Healthcare are significantly affecting patient waiting times, particularly in surgical settings (Gurumurthy *et al.*, 2021). Prolonged waiting time for patients in accessing timely surgeries is caused due to, among others, patient arrivals, incomplete pre-operative preparation, insufficient human resources, lack of collaboration between the departments, equipment availability and technological barriers (Bachelet *et al.*, 2019; Dawson *et al*., 2022). Healthcare organisations are facing challenges such as poor coordination between departments, doctors and other staff, inadequate resource management and unpredictable processes, which contribute to prolonged waiting times for patients (Drennan *et al.*, 2024). Financial constraints and process inefficiencies in maintaining well-equipped medical facilities contribute to delays in patient waiting time (Ebrahim *et al.*, 2025). These findings underscore the need for significant improvement in healthcare settings through enhanced resource management, workflow coordination and facility readiness. Technological barriers and resistance to adopting Lean 4.0 practices were observed as significant challenges in optimising surgical workflows, emphasising that AI integration requires careful change management and alignment with institutional capabilities (Abdallah and Alkhaldi, 2019). To streamline scheduling, predict bottlenecks and enhance decision-making in resource allocation, a strategic plan that incorporates Lean 4.0 principles into existing workflows is necessary (Rathnayake and Clarke, 2021). AI-driven technologies such as predictive analytics demonstrate high accuracy in predicting surgical delays, enabling relevant rescheduling to minimise patient waiting times (Choi *et al*., 2022). AI algorithms optimise clinic templates, estimate operating theatre lists and improve workforce allocation (Mahmoud *et al.*, 2021), thereby bridging the gap between expected and actual surgical durations while enhancing patient satisfaction and operational excellence (Dixon *et al*., 2024; Hamza *et al*., 2025; Ebrahim *et al*., 2025). Value Stream Mapping, 5S, Kaizen and Kanban, combined with AI-predictive analytics and real-time analytics, contribute to streamlining workflows in operating theatres (OT). At the Mayo Clinic, Value Stream Mapping, with predictive analytics, has reduced patient waiting times. These findings highlight that AI-driven tools shape scheduling management while lean simplifies workflows, enabling staff to predict demands based on medical records (Mahmoud *et al*., 2021). Healthcare firms can efficiently reduce patient waiting times by minimising waste through lean tools and leveraging AI for intelligent scheduling. The combination of Kaizen with AI tools, such as real-time resource allocation, significantly optimises inventory management and reduces delays (Abdallah and Alkhaldi, 2019; Ebrahim *et al*., 2025).

While Lean and AI offer efficiency gains, their impact is shaped by organizational context, staff readiness and data quality. Contradictions emerged where tools were available but underutilised due to fragmented systems, limited expertise or resistance to workflow changes. Effective implementation therefore requires leadership support, participatory change management and iterative piloting to translate technical capabilities into sustained operational improvements. For sustainable improvements, healthcare firms need to integrate Lean basics and AI in scheduling while providing staff training and involving them in planning and ensuring data accuracy (Mahmoud *et al*., 2021; Powell, 2024). Botha *et al*. (2024) emphasised that AI integration should start with a small dataset and gradually increase, highlighting the significance of incremental integration through small-scale pilots to minimise risks before scaling up.

The findings revealed that the future of AI and Lean lies in tracking staff shifts, managing sick leaves, doctor availability and equipment use that leads to better scheduling. Lean methods ensure standardisation and efficiency, and collectively optimise staff allocation, shifts and resources (Huang *et al.*, 2024). However, Botha *et al*. (2024) outlined that while AI-driven tools efficiently optimise resource allocation through predictive data analysis, they pose threats such as bias and discriminatory services. This suggests that integrating AI and lean implementation in healthcare can enhance scheduling and efficiency, but careful oversight is crucial to mitigate the risks associated with biased data. The findings are context-specific to operating theatres and may not fully apply to other healthcare settings. Notably, some interview accounts reveal tensions and contradictions in the effectiveness of Lean interventions. For instance, one participant reported that Lean implementation led to only “*one and a half minutes*” of time reduction in practice, which contrasts sharply with other accounts highlighting substantial efficiency gains from combined Lean–AI approaches. This discrepancy suggests that the impact of Lean is highly context-dependent (Guerrero *et al.*, 2025), influenced by factors such as staff engagement, adherence to standardised procedures, departmental culture and leadership support. It also highlights that the mere presence of Lean tools does not guarantee operational improvement; without sustained follow-through, visible leadership endorsement and integration with complementary technologies like AI, efficiency gains may be minimal. Such conflicting evidence underscores the importance of considering organisational readiness, micro-level workflow dynamics and human factors (Guimarães *et al.*, 2025) when interpreting the benefits of Lean 4.0 and cautions against assuming uniform effectiveness across diverse healthcare contexts.

## 6. Theoretical contributions

This study makes several important theoretical contributions to the literature on Lean management, AI integration and healthcare operations. Traditionally, Lean management theory emphasises waste reduction, process standardisation and workflow stabilisation (Abdallah and Alkhaldi, 2019). This study extends Lean theory by demonstrating that its effectiveness can be amplified through AI-driven predictive analytics and real-time decision-making in healthcare operations. The findings show that AI complements Lean principles by enabling anticipatory interventions such as predictive scheduling, dynamic resource reallocation and proactive monitoring of theatre readiness thereby transforming Lean from a reactive process improvement methodology into a dynamic, predictive framework for operational excellence. This contribution advances the theoretical understanding of Lean 4.0, positioning it as a hybrid model that combines traditional process efficiency tools with intelligent, data-driven capabilities. The study demonstrates that organizational culture, leadership commitment and staff engagement critically influence the success of Lean 4.0 interventions. This theoretical lens explains why similar Lean 4.0 interventions may yield variable results across different hospitals or teams.

The study provides empirical evidence for theories of resource coordination and workflow optimisation (Liu *et al.*, 2016). By illustrating how AI-driven predictive analytics, combined with Lean tools like 5S, Kanban and Value Stream Mapping, can compress delays, optimise theatre utilisation and enhance scheduling reliability, the research contributes to theoretical frameworks around efficient resource orchestration in complex, stochastic environments. The findings articulate mechanisms through which Lean stabilises processes while AI introduces flexibility and foresight, offering a more integrated conceptualisation of operational performance improvement. The research develops the concept of Lean 4.0 as a synergistic paradigm, in which Lean principles provide structure and standardisation (Javaid *et al.*, 2024), while AI provides predictive insight and proactive decision support. This theoretical contribution offers a framework for understanding the complementarities, boundary conditions and contingencies inherent in implementing AI-augmented Lean systems in healthcare settings. It positions Lean 4.0 not just as an incremental improvement tool, but as a strategic approach capable of aligning operational processes with dynamic, real-world demands.

## 7. Practical contributions

The findings of this study provide a specific roadmap for healthcare administrators to transition from traditional Lean to a “Lean 4.0” model in operating theatres. Our study data suggests four targeted areas for implementation. Practically, managers should move away from static daily lists and implement “*Proactive List Reshaping*.” We recommend that hospitals integrate these micro-efficiencies with Real-Time Analytics. For example, instead of manual handoffs, hospitals should use the real-time status alerts suggested by P5 to trigger cleaning and setup teams simultaneously, directly addressing the “interdepartmental fragmentation” noted by P2. *C) Hybrid Inventory Management (Digital Kanban):* Drawing from the Cleveland Clinic case study mentioned by P5, we recommend the integration of AI for real-time resource allocation alongside Lean's Kanban system. Practically, this means using AI to predict which surgical kits will be needed. To overcome the “cultural resistance” and “technological barriers” identified in our results, leaders should follow the “step-by-step” approach advised by P8 starting with AI for monitoring before moving to clinical decision-making, while involving staff in the planning to ensure data accuracy.

To help managers identify their current operational position and required next steps, we propose a Lean 4.0 Adoption Typology as described in Table 4. This matrix categorizes OT departments based on their Technical Integration (AI/Data) and Process Maturity (Lean/Culture), grounded in the specific insights of our participants.

**Table 4:** Lean 4.0 adoption typology

		Technical integration	
Process maturity		Low Technical Integration (Manual Data/Siloed Systems)	High Technical Integration (AI/Real-time Analytics)
	High Process Maturity (Standard Work/Kaizen Culture)	Quadrant 1 Lean Traditionalist	Quadrant 4 Lean 4.0 Exemplar
	Low Process Maturity (Fragmented/Unstandardized)	Quadrant 2: Reactive Firefighter	Quadrant 3: Digital Island

The reactive firefighter are hospitals which experience the “fragmented systems” and “late starts” described by P2. The priority here is basic “Systems Thinking” and Lean stabilization before introducing AI. The lean traditionalist has a strong Kaizen culture, as evidenced by P9's accounts of staff “saving minutes” through trolley and IV-line readiness. For these managers, the “85% predictive accuracy” of AI (as noted by P2) is the missing link to move from reactive to proactive scheduling. The digital island has invested in AI but face the “cultural resistance” and “technical difficulty” identified in Theme 1. As P8 suggests, the strategy must be to “train the staff and involve them in planning” to ensure the data is used in decision-making. The Lean 4.0 exemplar represents the “Future” envisioned by P7, where Lean and AI are “100% together”. This is exemplified by the Mayo and Cleveland Clinic cases cited by P5, where Lean's Kanban and VSM are supercharged by AI-driven real-time allocation.

For strategic self-diagnosis, managers can plot their organizations or departments current state to identify specific gaps.

## 8. Conclusion

This study proposes a phased implementation strategy, grounded in participants' experiences, beginning with small-scale pilot projects. Healthcare organisations should initially deploy basic Lean tools alongside AI applications for staff shift tracking, managing sick leave, monitoring physician availability and optimising equipment utilisation. Participants emphasised that starting small enables teams to adapt to workflow changes, ensures data accuracy for AI tools and demonstrates early gains, thereby building staff confidence and mitigating resistance.

Several methodological limitations warrant acknowledgement. The study employed a small, convenience sample of nine participants recruited via LinkedIn, which may limit the breadth of perspectives captured. Future research with larger, more diverse samples could strengthen the generalisability of these results.

Future investigations should adopt multi-site data collection across different hospital types and geographical regions would provide richer contextual variation and comparative insights. This expanded research agenda would provide policymakers and practitioners with comprehensive evidence for mandating Lean 4.0 approaches in healthcare systems.
